# Performance assessment of compression ground anchors in urban deep excavations through numerical and field investigations

**DOI:** 10.1038/s41598-025-24337-5

**Published:** 2025-11-18

**Authors:** Merve Akbas, Gokalp Demirci, Bekir Bugra Ozdemir, Recep Iyisan

**Affiliations:** 1 Department of Civil Engineering, Istanbul Technical University, Istanbul, 34485 Turkey; 2 On Ground Engineering and Construction LLC, Istanbul, 34722 Turkey

**Keywords:** Deep excavation stability, Compression-type anchors, Tension-type anchors, Finite element analysis, Field tests, Engineering, Natural hazards

## Abstract

This study evaluated the stability performance of compression and tension ground anchors used in deep excavations through numerical analyses and field tests. Compression anchors were observed to have advantages over tension-type anchors in terms of deformation control and safety factors. Under ultimate load conditions, the deformation of the compression anchors was recorded to be 14% lower. The safety factors for the anchors in clay soils were found to be nearly equal, both providing sufficient safety margins. Field tests demonstrated that compression anchors provide a broader load distribution, enhancing stability. These results support the use of compression-type anchors, particularly in urban projects.

## Introduction

In modern times, population growth and urbanization have necessitated more intensive construction in limited urban areas^1–5^. In particular, ensuring the safety of surrounding infrastructure and structures during construction has become a critical engineering challenge in projects involving multi-basement and high-rise buildings^6^. Ground movements and stresses occurring during deep excavations pose risks to surrounding structures, and controlling these effects has become a fundamental requirement in ensuring excavation stability^7–9^. In this context, various retaining systems are widely used to enhance excavation stability and environmental safety.

Retaining systems are designed to stabilize excavation surfaces by counteracting earth pressures behind them and generally consist of a combination of vertical and horizontal support elements. Vertical support elements in retaining systems include bored piles, diaphragm walls, micro-piles, soldier pile walls, shotcrete, and steel sheet piles, while horizontal support elements comprise ground anchors, soil nails, and steel struts. Among the horizontal support elements, prestressed ground anchors, which operate under axial tensile forces, are widely preferred due to their cost-effectiveness and applicability in various soil and rock conditions. However, the use of prestressed ground anchors operating under axial compressive forces as horizontal support elements in retaining systems is not as common. Therefore, more research is needed on the effects and limitations of different types of ground anchors on stability^6,10^.

**Fig. 1:**
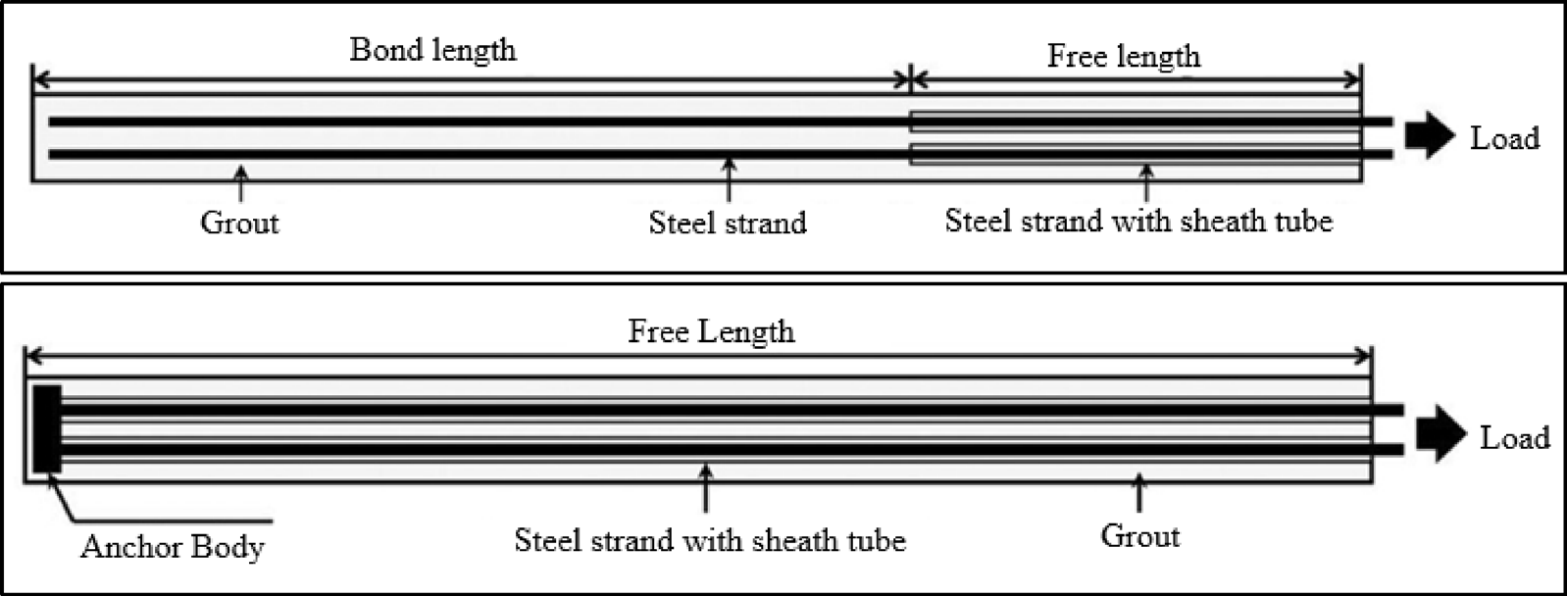
Schematic diagram of (**a**) tension (**b**) compression anchor^10^.

Traditionally used tensile ground anchors are widely employed horizontal support elements designed to work under axial tensile forces to ensure the stability of excavation support systems. As shown in Fig. 1(a), these types of anchors consist of two main components: a free length and a bonded length. The steel tendons in the free length are sheathed to prevent contact with grout, while the tendons in the bonded length are in adherence with grout, facilitating the transfer of applied loads to the bonded zone and enabling interaction with the ground^10^. However, tensile ground anchors have certain significant limitations. The brittle behavior of grout under tensile forces in the bonded zone of tensile anchors can lead to cracks, resulting in groundwater leakage and reduced resistance to corrosion^11^. The applicability of tensile ground anchors is restricted by the variety of soil layers, the magnitude of prestress loads, their service life, and their limited resistance to corrosion due to grout’s brittle behavior under tensile forces in the bonded zone^11–14^.

The limitations of tensile anchors have led to the development of compression ground anchors. Designed to operate under axial compressive forces instead of tensile forces, compression ground anchors overcome the limitations of tensile anchors and offer a broader range of applications. This design ensures a longer service life under high prestress loads^15,16^. As shown in Fig. 1(b), compression anchors consist of steel tendons encased in a polyethylene sheath to avoid contact with grout and structural elements, including an end plate to which the tendons are attached.

**Fig. 2:**
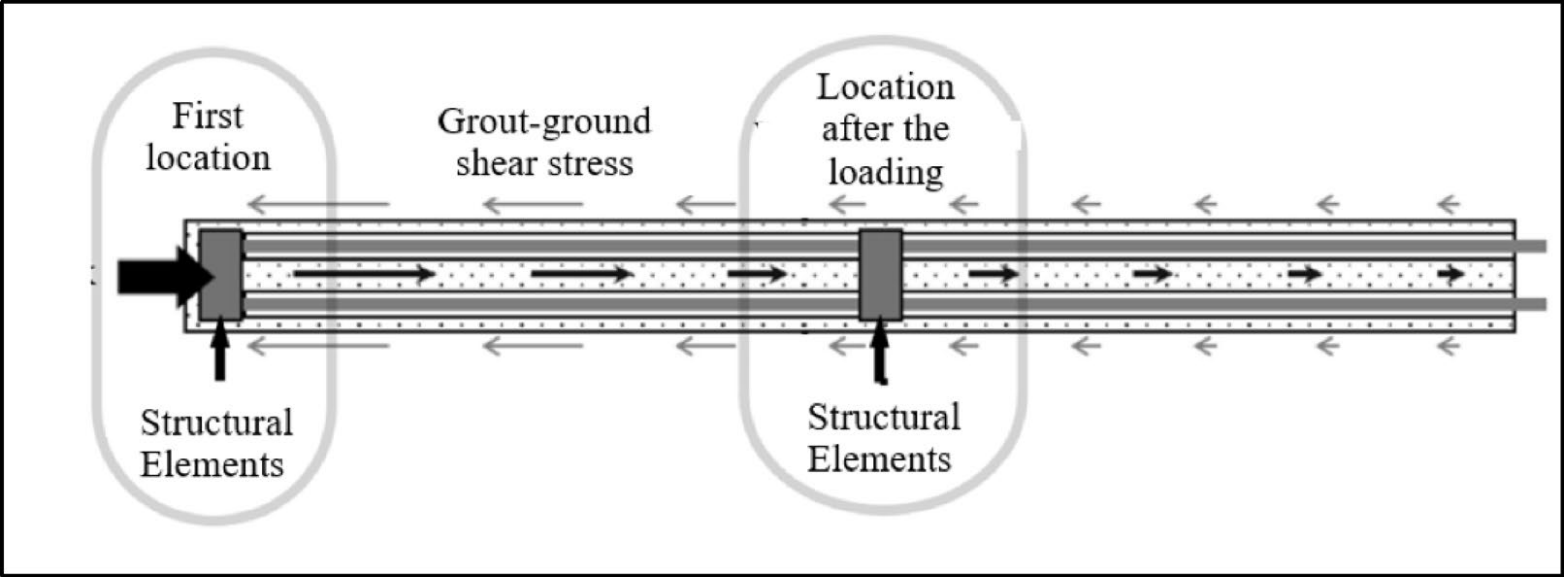
Schematic diagram of working principle of a compression anchor^20^.

The working principle of compression anchors is based on the transfer of the entire applied load to the structural element located at the end of the borehole through the sheathed tendons. During this process, shear stresses develop between the grout and the soil, and as the prestress load is gradually increased, the structural element at the end of the anchor borehole moves toward the vertical element of the retaining system, as illustrated in Fig. 2. This movement induces a compression force on the grout, resulting in the generation of shear resistance between the grout and the borehole wall, thereby enabling the active loading of the anchor^11,17–19^.

The working principle of compression anchors forms the basis of the advantages they provide over tensile anchors. In compression anchors, the operation of the grout under compression forces causes an expansion in the diameter of the borehole, thereby increasing the frictional resistance between the grout and the borehole wall. Furthermore, their high resistance to compressive forces enables the application of higher prestress loads to this type of anchor^17,18^. The sheathed structure of the steel tendons not only ensures high durability against corrosion but also allows the tendons to be easily unloaded and removed from the structural element in the event of decommissioning, facilitating their reuse in other projects. These features make compression anchors a durable and reliable option, particularly in environments near water or with high humidity. In this context, considering the limitations of tensile anchors, such as corrosion resistance, prestressing capacity, and reuse potential, investigating whether compression anchors can address these shortcomings has become a critical necessity. However, while the limitations of tensile anchors have been extensively examined in the literature, comprehensive studies evaluating the potential of compression anchors to overcome these limitations remain scarce^21^.

In this study, which compares the performance of compression and tensile ground anchors in terms of stability, deformation, and safety factors, the aim is to evaluate the application potential of compression anchors in constrained urban areas and highlight their advantages in enhancing excavation safety. Additionally, the study seeks to contribute to the literature by comprehensively demonstrating the superiority of compression ground anchors over tensile anchors and assessing the applicability of this emerging technology in the field of geotechnical engineering. To this end, the performance of compression and tensile ground anchors during deep excavations was examined using two primary methods: numerical modeling based on the finite element method and experimental investigations conducted under real field conditions. Anchor performance was evaluated based on key parameters such as deformation behavior, frictional forces arising from soil-anchor interaction, and safety factors. The strengths and weaknesses of both anchor types were identified, and their applicability in deep excavation projects was assessed. The findings demonstrate that compression anchors have significant potential for use in confined urban spaces and projects with high environmental risks, contributing to the safer and more sustainable implementation of deep excavation projects.

## Methods

In this study, a comprehensive evaluation was conducted to assess the performance of compressive and tensile ground anchors in ensuring stability during deep excavations. The investigation employed both numerical analyses and field experiments, providing a detailed understanding of anchor behavior under various conditions. By integrating numerical results with field data, the study aimed to offer reliable insights into the suitability and effectiveness of these anchor types in deep excavation projects, particularly in soils with relatively low bearing capacities, such as clay.

### Numerical modeling

The Finite Element Method (FEM) was utilized in the numerical analyses to evaluate the performance of compression and tensile anchors in deep excavation projects. These analyses were carried out using Plaxis 2D 2023 software, which enabled the detailed modeling of different anchor types within identical soil profiles. The primary objective was to investigate the interaction between the anchors and the surrounding soil, focusing on the distribution of loads and frictional forces at the grout-soil interface. Additionally, the analyses aimed to compare the deformation behaviors of anchors under varying load conditions.

#### Modeling parameters and material properties

**Fig. 3:**
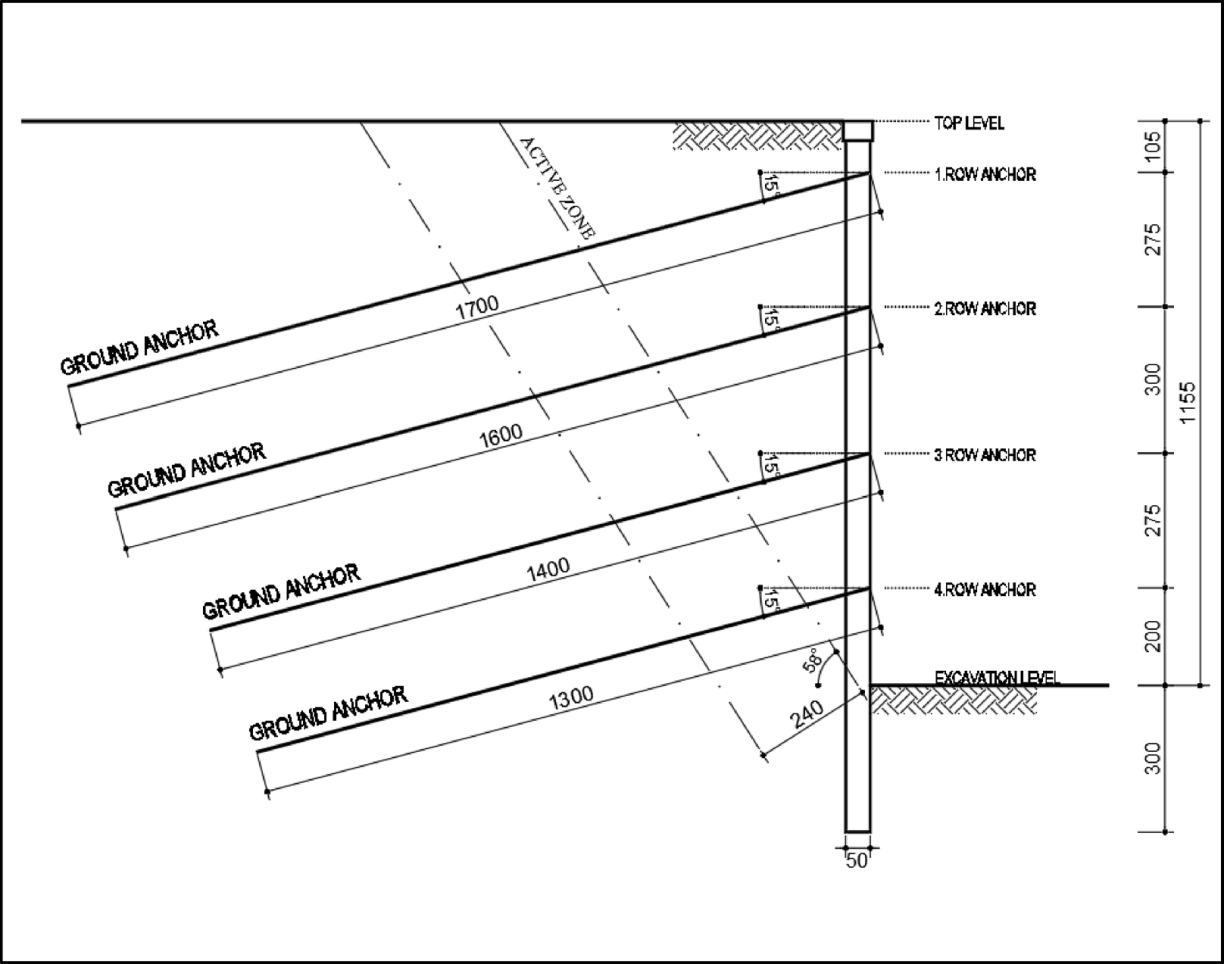
The cross-section view of the excavation support system.

The modeling parameters used in this study were based on widely accepted values in the literature and the standards outlined in Turkish regulations for excavation support structures^22^. As shown in Fig. 3, the excavation support system was designed as a bored pile system supported by four rows of anchors. The diameter of the bored piles was specified as 0.50 m, and their length was set at 14.55 m.

In the analyses, a homogeneous clay profile was adopted to represent the site conditions. The parameters in Table 1 were derived from the site investigation data presented in Table 5, which included SPT tests, undisturbed sampling, and laboratory measurements. While the actual site conditions include fill, sandy clay, and silty clay layers, the dominant silty clay stratum governs the anchor–soil interaction, and its representation as a homogeneous profile provided a conservative and consistent modeling framework. These values were consolidated and calibrated to reflect the dominant silty clay stratum, which governs the deformation behavior of the excavation. The stiffness and strength parameters were adjusted in accordance with the^22^ and validated against laboratory test results. This calibration ensured consistency between the site data and the numerical model, while allowing the anchor performance comparison to remain focused on the effect of anchor type rather than soil heterogeneity.

The elastic properties, cohesion, and internal friction angle parameters of the soil materials were determined based on field data and defined in the Plaxis 2D software. The Hardening Soil Model (HSM) was employed as the constitutive relation for the fill, sandy clay, and silty clay layers. This choice was made to maintain consistency across the soil layers and to enable a more realistic representation of stress-dependent stiffness compared to the Mohr–Coulomb model. The HSM has been widely applied to a variety of soil types in previous studies^4,7,8^. While alternative constitutive models could have been selected for individual soil layers, using a single model ensures comparability of results and allows the study to focus on the relative performance of compression and tension anchors without bias from model variability.

In the project site where field tests were conducted, borehole investigations did not reveal the presence of groundwater; therefore, the effects of groundwater were not considered in the numerical analyses. The grout–soil interface was modeled using an interface reduction factor (Rinter), which governs the mobilized shear strength at the contact. For the clay profile used in numerical analyses, Rinter was taken as 0.85, consistent with Turkish excavation support standards^22^. For sandy clay and silty clay layers in the field test section, grout–soil interface shear strength was defined as τ_interface_ = Rinter × (c′ + σ′ tan φ′). This approach provides a realistic representation of the interaction and ensures consistency across the soil profiles considered. In the finite element program, bored piles with a diameter of 0.50 m, spaced at 0.85 m intervals horizontally, were modeled as “plate” structural elements to simulate the bending resistance and axial stiffness of the beams. The material properties of the bored piles are summarized in Table 2.

**Table 1:** Geotechnical properties of clay.

Properties	Symbols	Units	Clay
Material model	–	–	Hardening soil
Material type	–	–	Drained
Unit weight	γ	kN/m^3^	20
Deformation modulus ^1^	E_50_^ref^	MN/m^2^	70
Deformation modulus ^2^	E_oed_^ref^	MN/m^2^	70
Deformation modulus ^3^	E_ur_^ref^	MN/m^2^	210
Poisson ratio	ν_ur_	–	0.2
Cohesion	c′_ref_	kN/m^2^	25
Friction angle	ϕ	°	25
Dilatation angle	Ψ	°	0

^1^Reference stiffness for primary loading.

^2^Plastic straining due to primary compression.

^3^Reference stiffness for unloading and reloading.

**Table 2:** Material properties of pile.

Properties	Symbols	Units	Pile
Material model	–	–	Elastic
Specific weight	w	kN/m/m	7.5
Normal stiffness	EA	kN/m	7.39 × 10^6^
Bending stiffness	EI	kN/m^2^/m	115.50 × 10^3^
Poisson ratio	ν	–	0.15
Thickness of equivalent wall	d	m	0.4331

#### Anchor design

The compression and tension-type ground anchors used in the model were evaluated with varying lengths and dimensions, aiming to conduct a detailed comparison of performance differences among these anchors. For tension-type anchors, different cement adhesion values were defined for the free and bonded zones, and the steel tendons were modeled to prevent contact with cement in the free zone through sheath coating. In compression anchors, the tendons were similarly sheathed to avoid contact with cement, and prestressing force was transferred to the structural element located at the tip.

In tension anchors, only the free zone was sheathed, whereas in compression anchors, the tendons were sheathed along their entire length. These tendons were modeled in the finite element program as a “node-to-node anchor” structural element, which is a two-node elastic spring capable of withstanding both tensile and compression forces. It was assumed that the tendons exhibited elastoplastic behavior, as the applied prestressing force could cause permanent deformations if it exceeded a certain threshold and was subsequently removed. The material properties of the steel tendons used in the numerical analyses are summarized in Table 3.

**Table 3:** Material properties of steel tendons.

Properties	Symbols	Tension anchor	Compression anchor
Material type	–	Elastoplastic	Elastoplastic
Spacing	L_s_	3.40	3.40
Normal stiffness	EA	84 × 10^3^	84 × 10^3^
Maximum tensile force	|F_max, tensile_|	780	780
Maximum compression force	|F_max, compression_|	780	780

To represent the frictional interaction between the grout and soil in ground anchors, an “embedded beam row” structural element was used in the numerical model. This element was assumed to exhibit elastic behavior in the bonded zone for tension anchors and along the entire length for compression anchors, reflecting their respective load transfer mechanisms. The material properties of the grout, essential for these interactions, are summarized in Table 4.

**Table 4:** Material properties of Grout in clay.

Properties	Symbols	Units	Tension anchor	Compression anchor
Material type	–	–	Elastic	Elastic
Unit weight	γ	kN/m^3^	20	20
Diameter	D_anchor_	m	0.135	0.135
Deformation modulus	E	kN/m^2^	14 × 10^6^	14 × 10^6^
Characteristic bearing capacity	T_k_	kN/m	50	50

#### Loading conditions

**Fig. 4:**
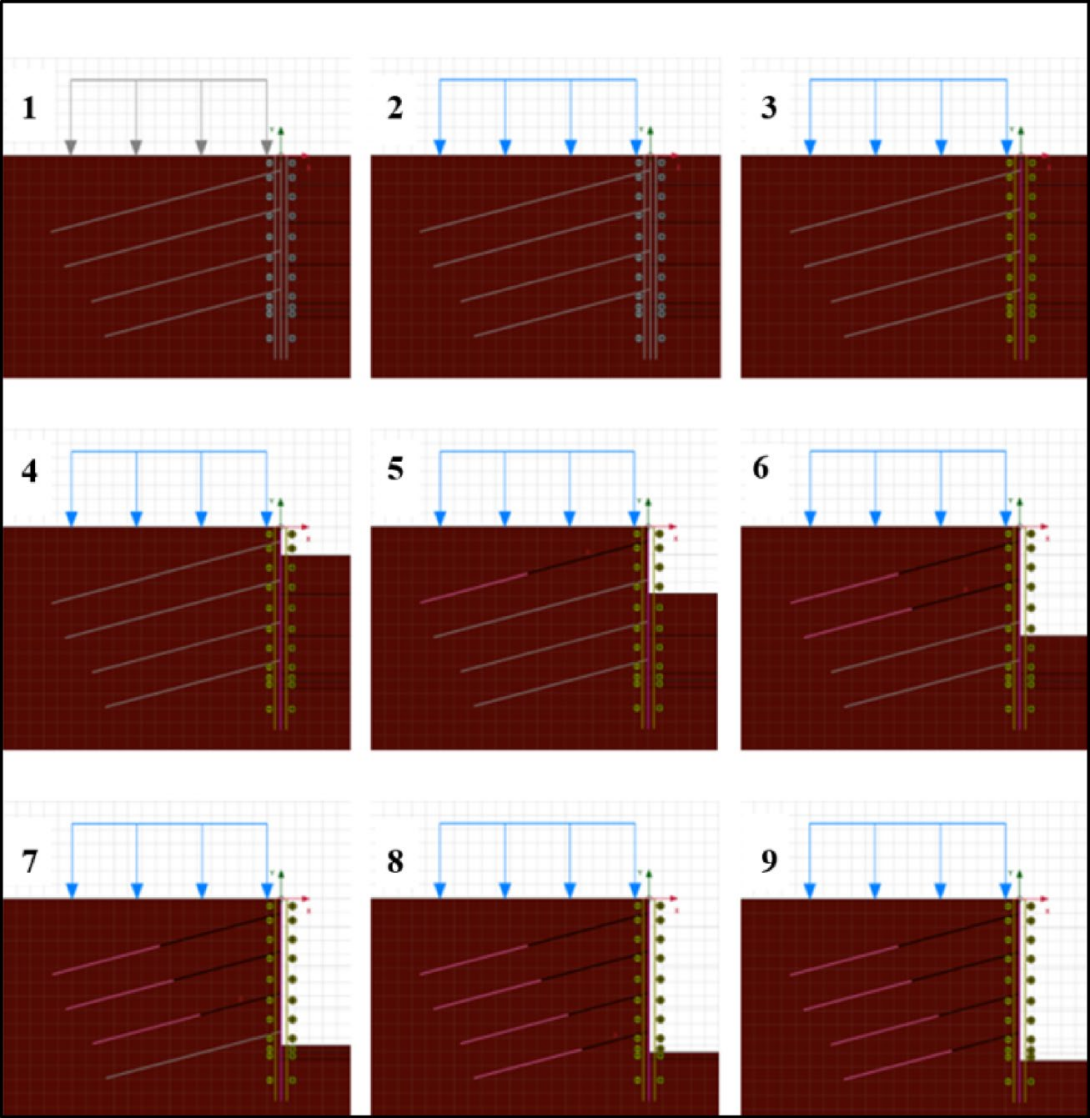
Finite element mesh and analysis stages.

In this study, the behavior of anchor systems under different loading conditions was analyzed by applying incremental loading. Specific loading conditions were incorporated into the finite element model for both anchor types, and boundary conditions were considered from a stability perspective. During loading, deformations of the anchor grout body, soil-anchor interactions, and the horizontal deformations of the retaining system were examined. Additionally, safety factors against global failure were calculated during the analyses. After defining the material properties in the finite element program, a geometric model was created, including soil layers, horizontal and vertical excavation support systems, and surrounding surcharge loads (assumed to be 15 kN/m^2^ due to the absence of surrounding structures). The finite element mesh was generated using triangular elements. Preliminary convergence and sensitivity checks were performed by varying mesh density, number of elements, and boundary extents. The results indicated that the difference in displacements was less than 2.5% between medium and fine meshes, while excessive computation time was required for the fine mesh. Therefore, a medium mesh size was adopted. The boundaries were extended sufficiently to eliminate edge effects, and further extension resulted in negligible changes (< 1%) in the outcomes. These checks confirm that the results are not significantly influenced by modeling constraints. The staged analysis in Fig. 4 represents the construction of the support system and excavation process.

### Field tests

For the field tests, a suitable site was selected in Bagcilar district, Istanbul. The soil properties of this area were analyzed in detail by drilling nine boreholes to a depth of 35.00 m. Standard penetration tests, groundwater level measurements, and undisturbed sample collection for laboratory testing were conducted. In the boreholes, the thickness of the fill layer varied between 1.00 m and 6.50 m, while the sandy clay layer was observed to reach thicknesses of up to 7.00 m in some boreholes. The thickness of the silty clay layer varied between 27.00 m and 31.00 m across all boreholes. The geotechnical parameters of the soil layers, as determined from a review of the literature and the results of field and laboratory tests, are presented in Table 5.

**Table 5:** Geotechnical parameters of layers.

Properties	Units	Fill	Sandy Clay	Silty Clay
Material type	–	Hardening soil	Hardening soil	Hardening soil
Material type	–	Drained	Drained	Drained
γ	kN/m^3^	17	17	20
E_50_^ref^	MN/m^2^	10	30	100
E_oed_^ref^	MN/m^2^	10	30	100
E_ur_^ref^	MN/m^2^	30	90	300
ν_ur_	–	0.2	0.2	0.2
c′_ref_	kN/m^2^	5	5	25
ϕ	°	25	25	28
Ψ	°	0	0	0

#### Preparation and installation of test anchors

**Fig. 5:**
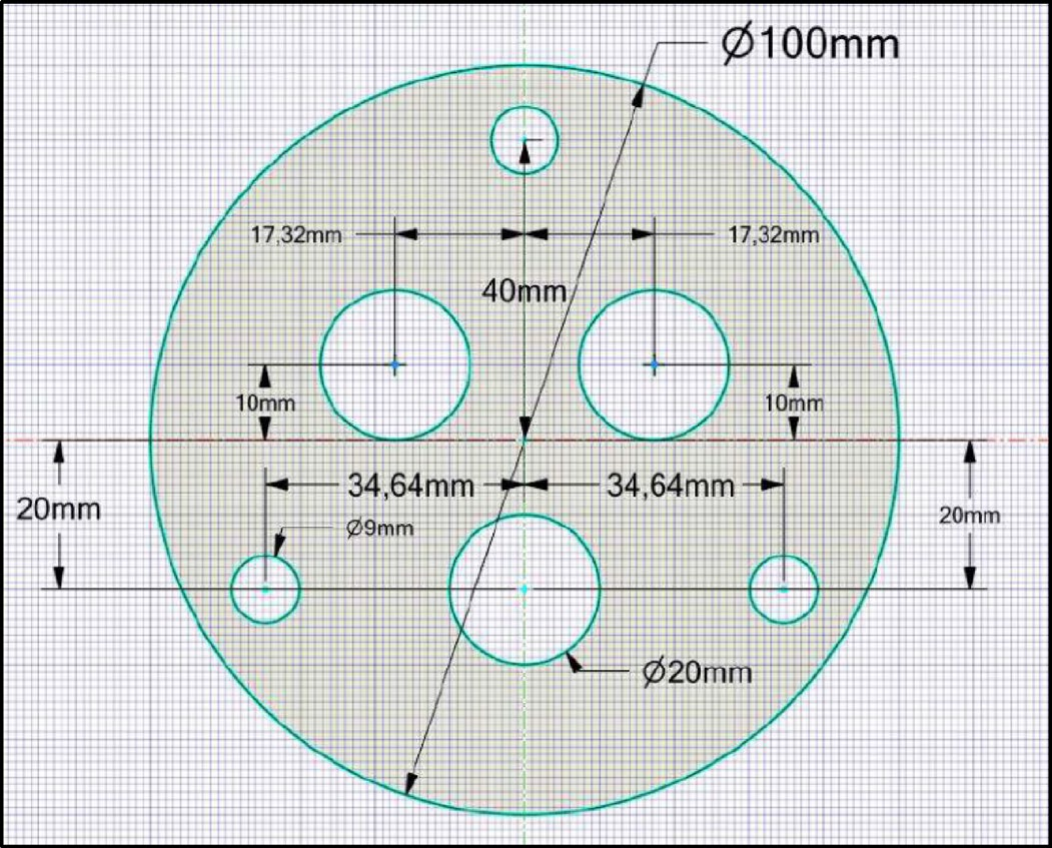
Anchor body geometric properties.

Anchors with specifications suitable for the test site were installed and prestressed for both anchor types. In compression anchors, the steel tendons were sheathed to prevent adhesion with cement and were connected to a structural element located at the anchor tip, as shown with dimensions in Fig. 5.

**Fig. 6:**
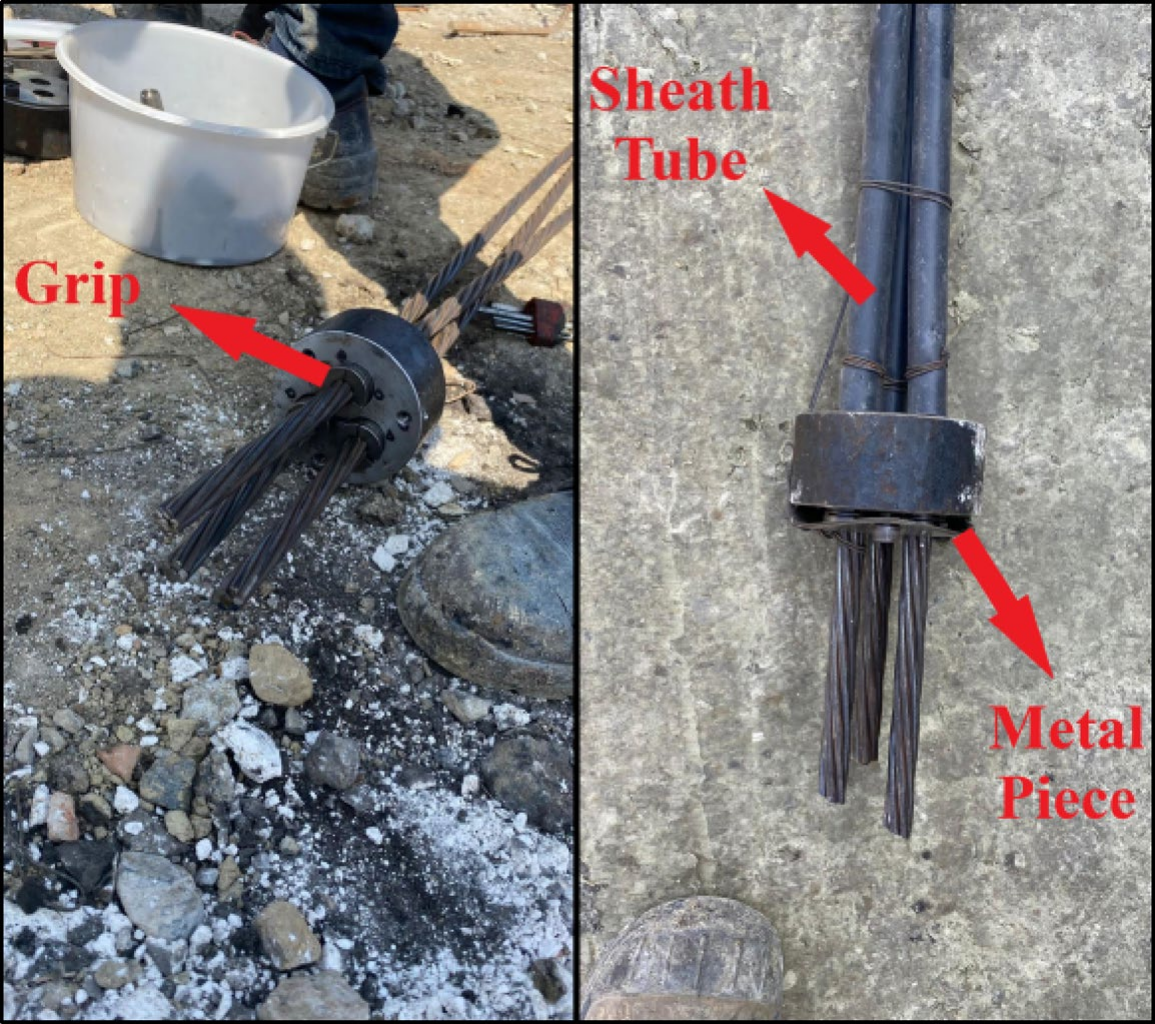
Components of 3 × 0.6” compression anchor.

In tension anchors, cement adhesion was established in the bonded zone. The properties of the tendons and the manufacturing details of the anchors used in the field tests are summarized in Tables 6 and 7, respectively. In compression test anchors, during the tensile testing phase, anchor grips were used in the structural element’s holes where the tendons were placed, as shown in Fig. 6 (photograph taken by the authors during the field test), to prevent the tendons from slipping out of the structural element towards the borehole opening when the prestressing force was applied. Additionally, to ensure that the grips did not slip out of the hole towards the bottom of the borehole during the insertion of the sheathed tendons, a circular steel plate with a thickness of 2 mm was bolted to a non-load-bearing structural element.

**Table 6:** Steel tendons properties of test anchors.

No	Test anchor type	Strand type	E (MN/m^2^)	A_strand_ (mm^2^/strand)	A_anchor_ (mm^2^)	*P*_t, k_ (kN/strand)	*P*_t, k_ (kN)
1	Tension	3 × 0,6”	200,000	140	420	260	780
2	Compression	3 × 0,6”	200,000	140	420	260	780
3	Compression	3 × 0,6”	200,000	140	420	260	780
4	Compression	3 × 0,6”	200,000	140	420	260	780

**Table 7:** Properties of test anchors.

No	Test anchor type	Drill diameter (m)	Length (m)	Free length (m)	Bond length (m)
1	Tension	0.135	16	8	8
2	Compression	0.135	16	16	–
3	Compression	0.135	14	14	–
4	Compression	0.135	14	14	–

For the test anchors, drilling operations with a diameter of 0.135 m were performed using a drilling machine. The residual debris and slurry within the boreholes were cleaned using an air compressor, preparing the boreholes for grouting. The grouting process was carried out using a tremie pipe, starting from the bottom of the borehole and filling up to the opening. The cement used for grouting was of the CEM I 42.5 R class, with a water-to-cement ratio of 0.45. The 28-day compression strength of the cement was determined to be 42.5 MN/m^2^.

#### Tensile tests and data collection

**Fig. 7:**
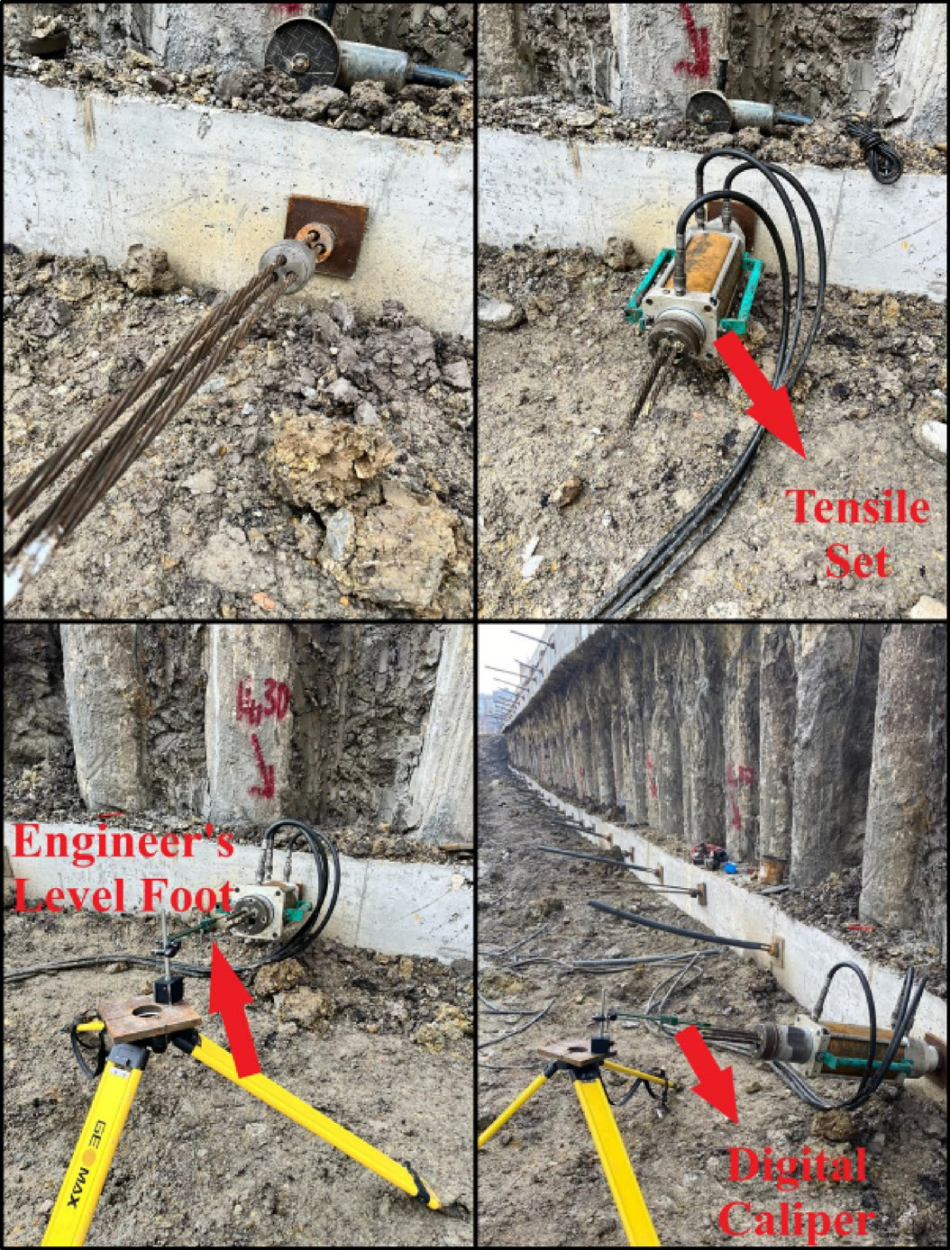
Instruments of tensile test.

Tensile tests were conducted to determine the deformation behavior of the anchors. Before performing the tensile tests, a waiting period of 7 days was observed to ensure that the grout achieved a minimum compression strength of 21 MPa^23^. During the tensile tests, the deformations occurring in the test anchors were recorded using a digital caliper, a precise measurement device fixed to the leveling foot shown in Fig. 7 (photograph taken by the authors during the field test). The prestressing process during the tensile tests was incrementally increased in accordance with the purpose of the experiments. At each load stage, the elongation of the tendons was measured and compared with their theoretical elongation values. These measurements were used as references to evaluate the accuracy and reliability of the numerical analyses. The load transfer in the bonded zone of tension anchors and the load transfer at the tip of compression anchors were detailed, and the behaviors of both systems were compared.

## Results and discussion

In this study, the deformation behaviors and safety factors of different anchor types were first examined through numerical analyses, followed by an evaluation of the load-bearing capacities and elongation values of compression and tension-type anchors under actual soil conditions through field tests.

### Numerical analysis results

**Fig. 8:**
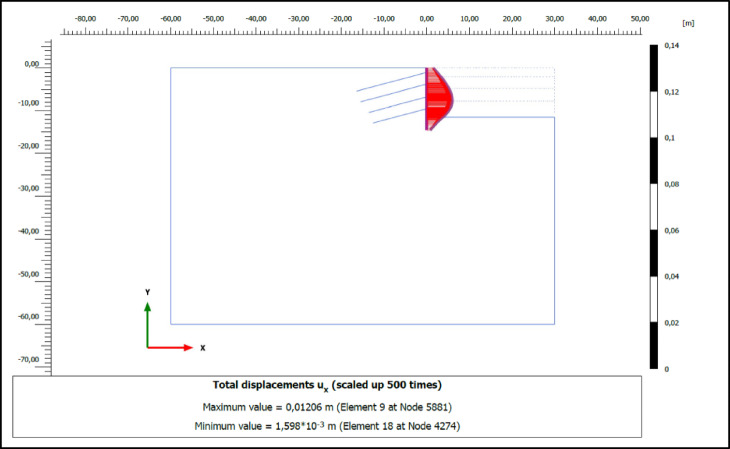
Horizontal deformation of excavation support system for compression anchors.

**Fig. 9:**
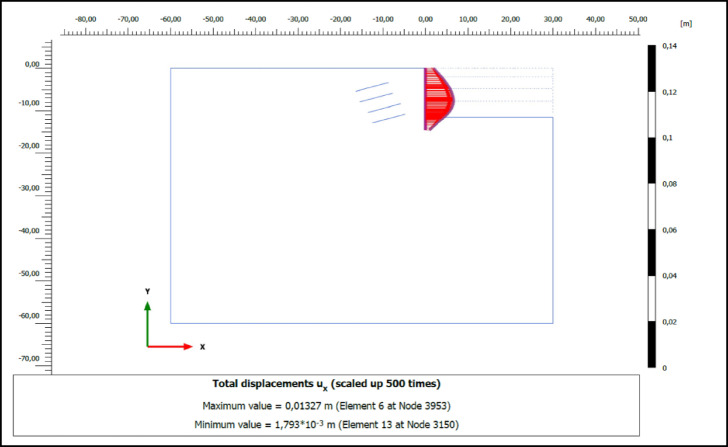
Horizontal deformation of excavation support system for tension anchors.

The performance of compression and tension-type ground anchors used for ensuring stability in deep excavations was analyzed using the finite element method in Plaxis 2D 2023 software. The horizontal deformations of the vertical support elements at the final excavation level in retaining systems employing compression and tension-type ground anchors are shown in Figs. 8 and 9, respectively. According to the analysis results, the horizontal deformation (u_x_) of the vertical support element in the system with compression anchors was determined to be 1.21 cm, while in the system with tension anchors, this value was found to be 1.33 cm.

**Fig. 10:**
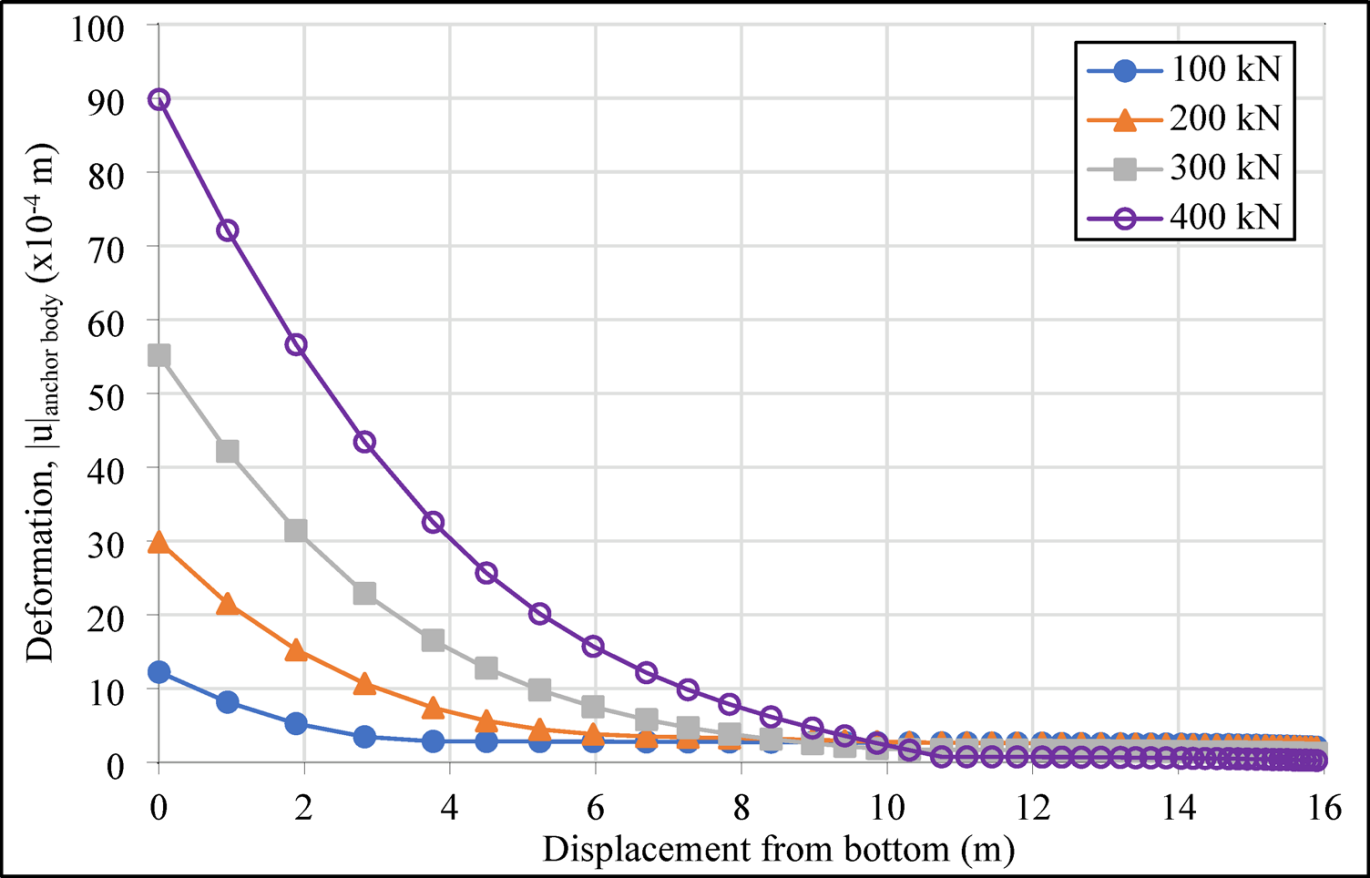
Deformation graph of the grout body of the compression anchor in the second row.

**Fig. 11:**
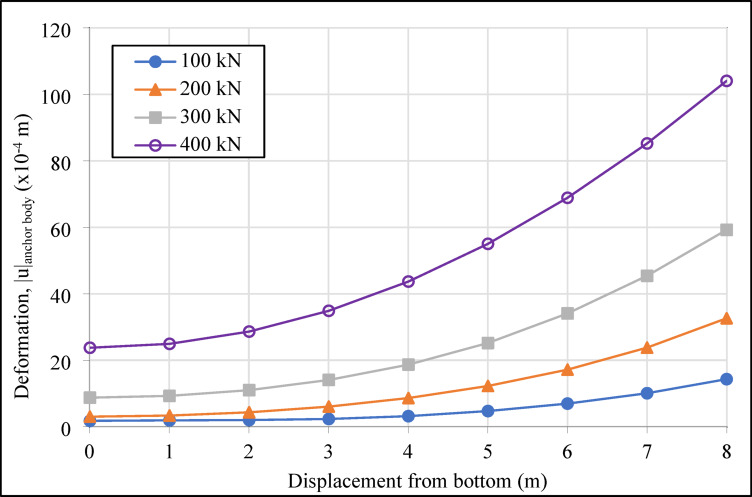
Deformation graph of the grout body of the tension anchor in the second row.

In the system designed with four horizontal support elements in the vertical direction, the grout deformation values (|u|_grout_) were determined as 1.51, 1.53, 1.48, and 1.16 cm from top to bottom for compression anchors, and 1.83, 1.87, 1.49, and 1.62 cm for tension anchors. The variation in deformation values in the grout body, under incremental loading on the compression and tension-type ground anchors with a drilling length of 16.00 m located in the second row of the analyzed retaining system, is presented graphically in Figs. 10 and 11, respectively.

As shown in Figs. 10 and 11, the maximum deformations in the grout body of the compression anchors under loading conditions were recorded as 12.22 × 10^-4^ m at 100 kN, 29.86 × 10^-4^ m at 200 kN, 55.17 × 10^-4^ m at 300 kN, and 89.83 × 10^-4^ m at 400 kN. For the tension anchors, the corresponding deformation values were 14.32 × 10^-4^ m, 32.64 × 10^-4^ m, 59.25 × 10^-4^ m, and 104.07 × 10^-4^ m. The results indicate that the compression anchors exhibited lower deformation values compared to the tension anchors at all load stages. For instance, under a 400 kN load, the deformation in the grout body of the compression anchor was approximately 14% lower than that of the tension anchor. This difference demonstrates that compression anchors provide higher rigidity in soil-anchor interaction, effectively limiting soil movements around the excavation area. In the analyses, the approximately 21% reduction in deformation provides a critical advantage, particularly in ensuring the safety of existing structures in densely populated urban projects. The higher deformation observed in the grout body of tension anchors under loading demonstrates that these systems are less effective in terms of deformation control.

These findings are consistent with the results of Zhang et al.^19^, who conducted a study in silty clay soils. In that study, the load-bearing capacity and structural element deformations of compression ground anchors were evaluated through tensile tests. For a two-tendon compression ground anchor, the load-bearing capacity was determined to be 260 kN, with a maximum structural element deformation of 300 mm. The evaluation highlighted that the advantages of compression anchors in providing homogeneity and rigidity in load transfer enhance their load-bearing capacity and limit deformations.

**Fig. 12:**
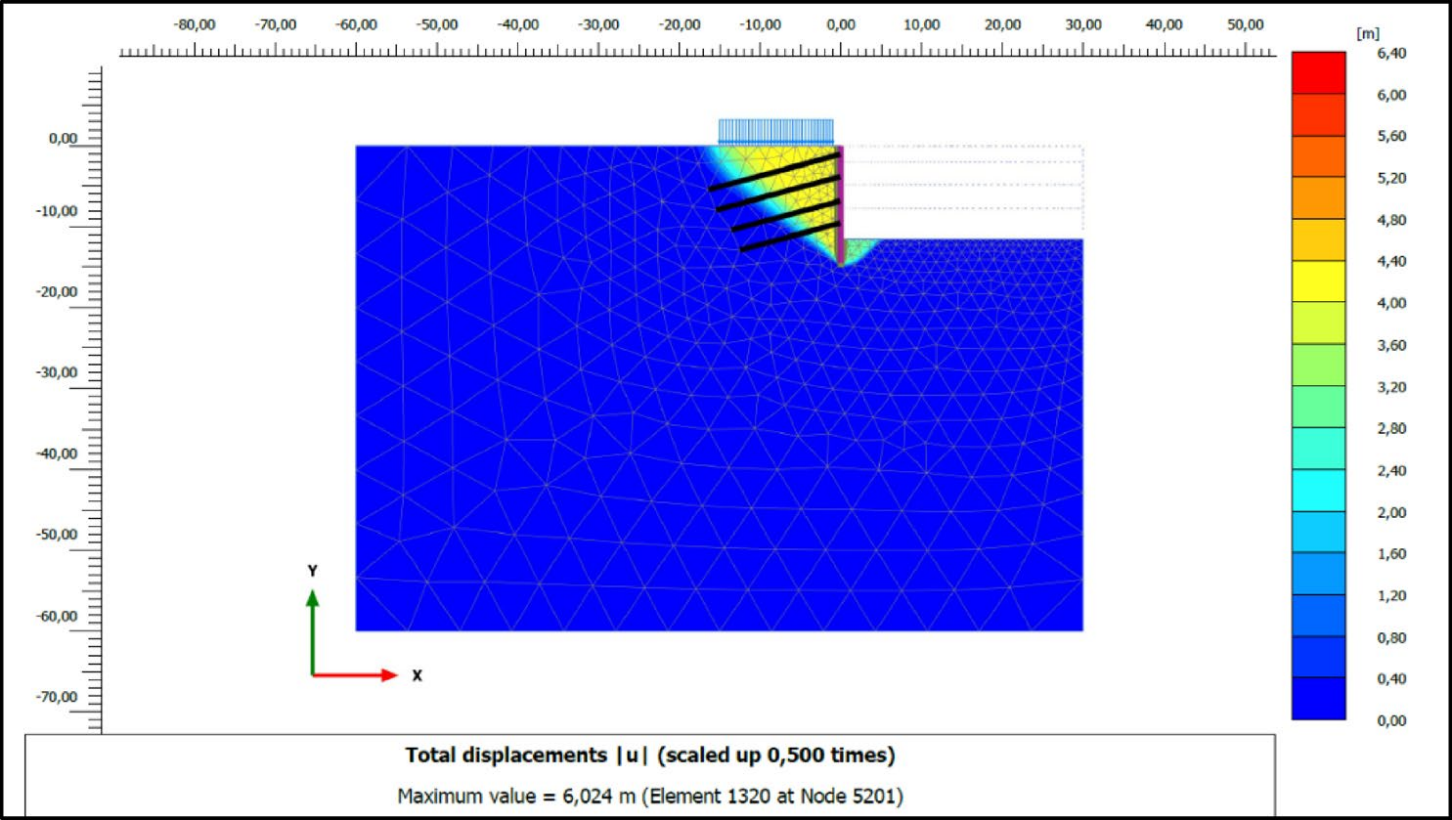
Slip surface of excavation support system for compression anchors.

**Fig. 13:**
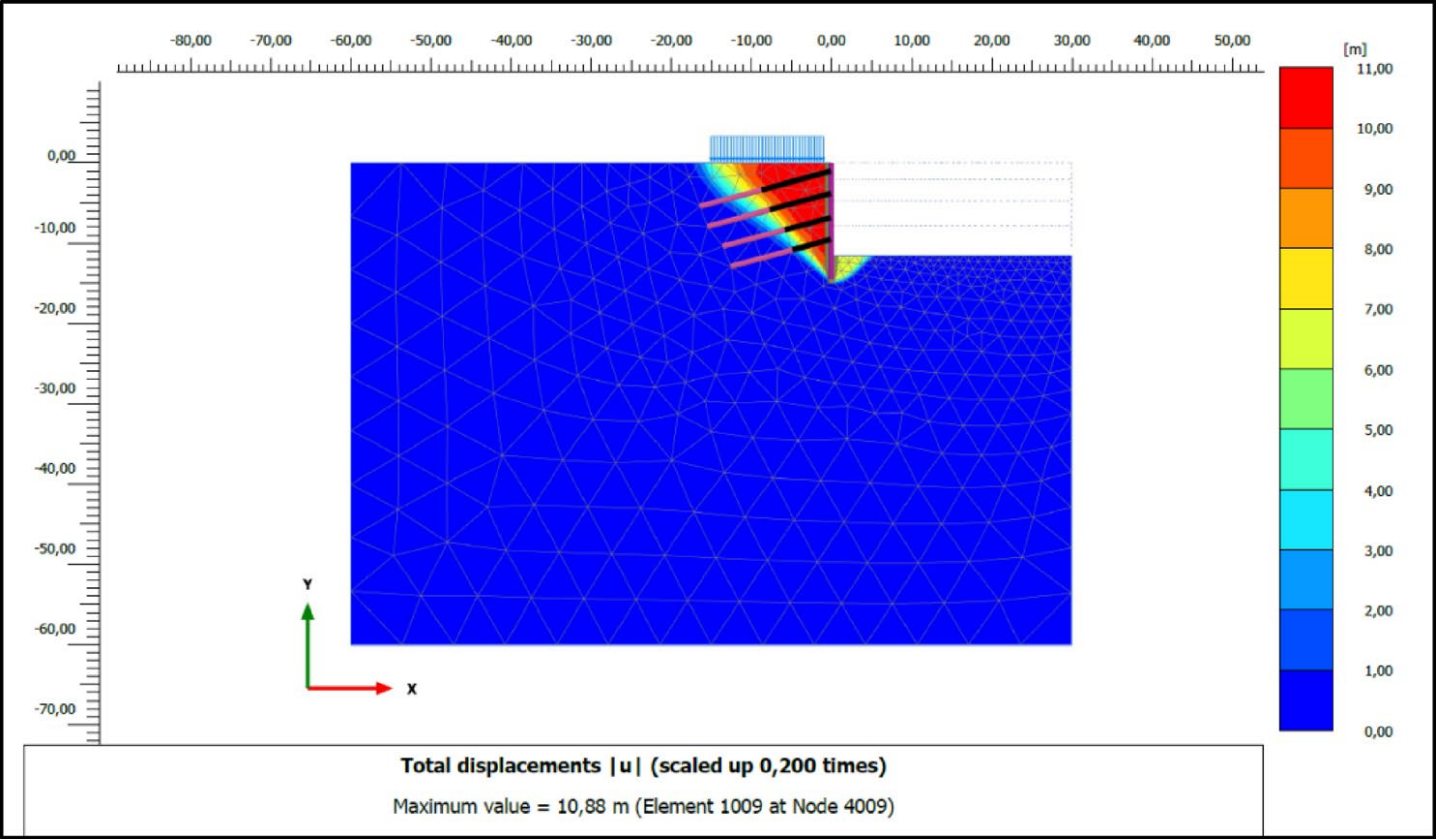
Slip surface of excavation support system for tension anchors.

Safety factors are a crucial metric for assessing the effectiveness of deep excavation support systems in ensuring overall stability and preventing failure. The safety factors obtained from numerical analyses indicate that the system with compression anchors has a higher safety factor against global failure compared to the system with tension anchors. In clay soils, the safety factor was determined to be 1.51 for compression anchors and 1.49 for tension anchors highlighting a marginally reduced capacity to maintain stability. The data demonstrate the potential of compression anchors to enhance stability, particularly in deep excavation projects conducted in clay soils. Similarly, Kim^24^ conducted a study comparing the safety factors and load transfer mechanisms of tension and compression ground anchors. A numerical model was developed to analyze the loads in the tendons, forces in the grout body, frictional forces at the grout-soil interface, and load transfer distributions. The results of the study showed that compression anchors provide a more balanced load transfer distribution and exhibit higher shear resistance. The failure surfaces formed during global failure in systems utilizing compression and tension anchors analyzed in clay soils are shown in Figs. 12 and 13, respectively.

**Fig. 14:**
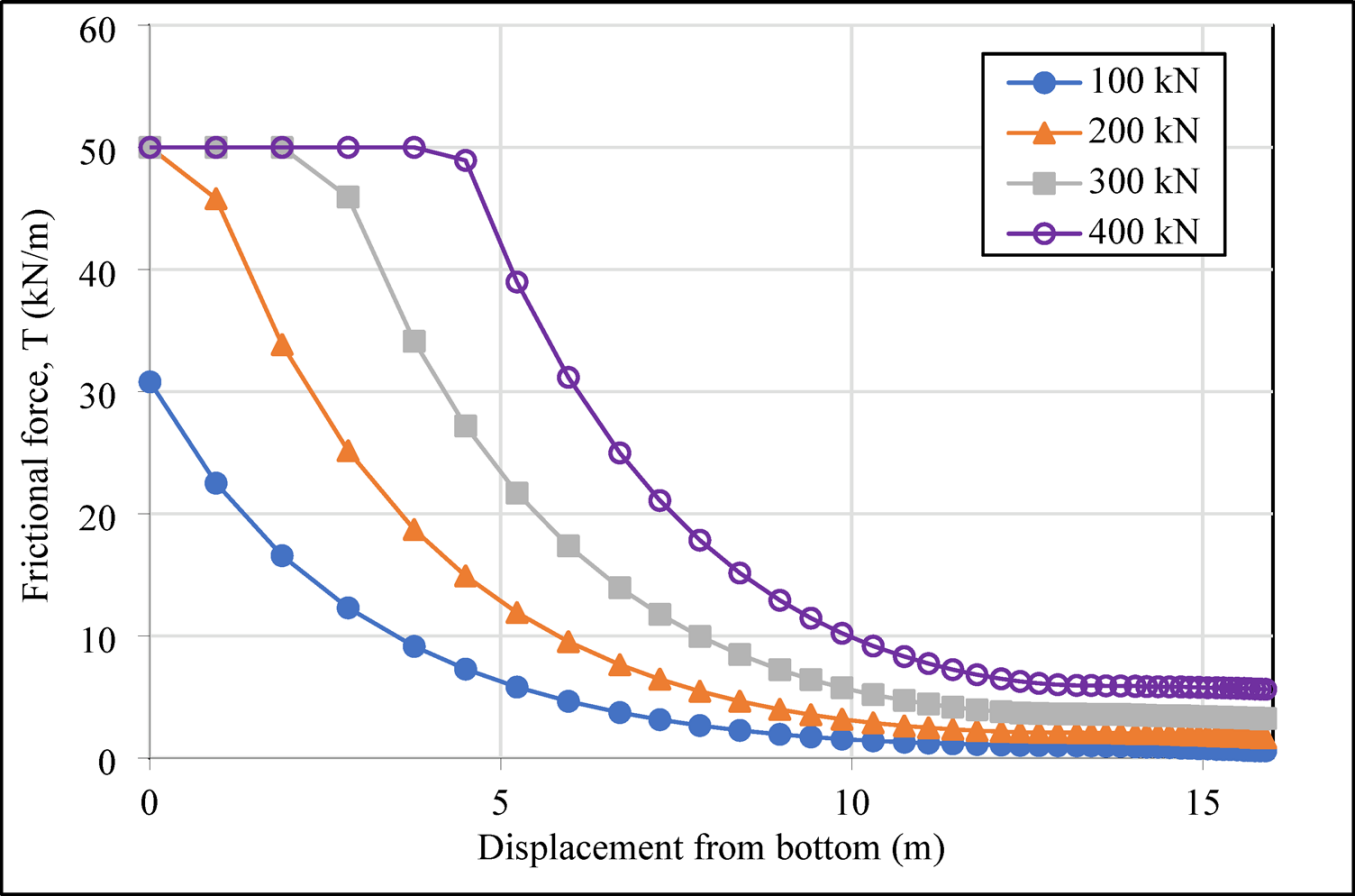
Frictional force values graph of compression anchor in the second row.

**Fig. 15:**
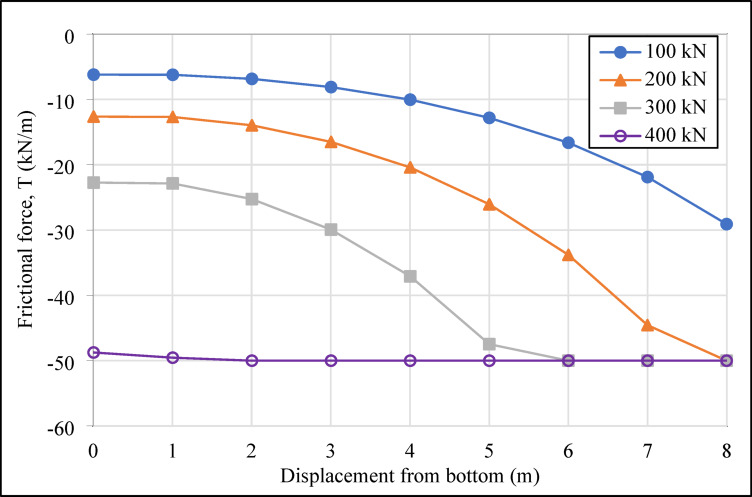
Frictional force values graph of tension anchor in the second row.

In the numerical analyses, the soil-anchor interaction and load distribution were examined for both anchor types, and the frictional forces between the grout and the soil wall were determined. The variation in frictional forces along the grout body under incremental loading for compression and tension ground anchors located in the second vertical row of the retaining system is shown in Figs. 14 and 15, respectively.

As shown in Fig. 14, under the initial load of 100 kN, the maximum frictional force was calculated as 30.79 kN/m for the compression anchor and 29.09 kN/m for the tension anchor. At subsequent loading stages, the maximum frictional force for both compression and tension anchors reached a final frictional force value of 50.00 kN/m. The maximum frictional force in compression anchors is concentrated at the end of the anchor hole, near the structural element region, during the initial loading condition (100 kN). This can be attributed to the direct transfer of the entire load to this region through the sheathed steel tendons. As the prestressing load is incrementally increased (200 kN, 300 kN, and final loading conditions), the final frictional force was observed to distribute from the structural element toward the anchor head, due to the formation of compression cracks at the end of the anchor borehole. This load distribution mechanism demonstrates a more balanced load transfer in compression anchors. In contrast, as illustrated in Fig. 15, the maximum frictional force for tension anchors is concentrated in the bonded zone, specifically at the point closest to the vertical element of the retaining system, during the initial loading condition (100 kN). However, as the load increases, this concentration leads to increased load losses and negatively impacts stability by causing cracks. Consequently, the final frictional force spreads toward the end of the anchor hole. These results indicate that, in clay soils, compression anchors demonstrate a significant advantage in load distribution, effectively controlling soil movements and contributing to the safety of the surrounding environment.

### Field test results

Field tests were conducted to validate the results of numerical analyses and to evaluate the performance of compression and tension-type ground anchors under actual site conditions. These tests were performed at a project site in Turkey with a clay soil profile, with a total of four tests conducted for each anchor type. The field tests were meticulously carried out to highlight the performance differences between these two anchor types in ensuring soil stability in the excavation area.

Tensile tests were conducted to measure horizontal and vertical deformations under the prestressing loads specified for both anchor types. During the tests, the deformations occurring in both anchor types were recorded using precise measuring devices. The deformation data were collected with high accuracy during the field tests and compared with the results of the numerical analyses. The test anchors included a single 0.6-inch tendon with a characteristic tensile strength of 260 kN, utilizing three 0.6-inch tendons in total. Since the test anchors consisted of three 0.6-inch tendons, the final load in the tensile test was determined to be 400 kN, based on the criterion that the prestressing load on a single tendon in the anchor bundle must not exceed 60% of its characteristic tensile strength^22^. During the tests, the elongation of the tendons was recorded at each load stage and compared with both theoretical predictions and numerical results. The measured elongations for compression anchors were higher than the theoretical values, primarily due to the load-transfer mechanism of compression anchors, which mobilize shear resistance over a broader zone compared to the bonded length in tension anchors. Additional factors such as heterogeneity of the soil layers, grouting quality, and early-age grout behavior further contributed to the observed differences. These findings are in line with prior studies^10,13,16^, which similarly reported that field measurements of compression anchor displacements tend to exceed theoretical predictions. These discrepancies highlight the ductile and distributed nature of compression anchor performance and do not affect the overall conclusions of the study. The theoretical elongation of the anchor tendon during the tensile test was calculated using Eq. (1).

1 $$\:\text{T}\text{h}\text{e}\text{o}\text{r}\text{e}\text{t}\text{i}\text{c}\text{a}\text{l}\:\text{E}\text{l}\text{o}\text{n}\text{g}\text{a}\text{t}\text{i}\text{o}\text{n}=\text{L}\text{o}\text{a}\text{d}\bullet\:\frac{\text{A}\text{n}\text{c}\text{h}\text{o}\text{r}\:\text{F}\text{r}\text{e}\text{e}\:\text{L}\text{e}\text{n}\text{g}\text{t}\text{h}+\text{J}\text{a}\text{c}\text{k}\:\text{L}\text{e}\text{n}\text{g}\text{t}\text{h}}{\text{S}\text{t}\text{r}\text{a}\text{n}\text{d}\:\text{A}\text{r}\text{e}\text{a}\bullet\:\text{S}\text{t}\text{r}\text{a}\text{n}\text{d}\:\text{N}\text{u}\text{m}\text{b}\text{e}\text{r}\bullet\:\text{E}\text{l}\text{a}\text{s}\text{t}\text{i}\text{c}\:\text{M}\text{o}\text{d}\text{u}\text{l}\text{u}\text{s}}$$

The elongation values obtained for Anchor 1 (tension type) and Anchors 2, 3, and 4 (compression type) under different load stages were analyzed and compared. All data are summarized in Table 8.

**Table 8:** Geotechnical parameters of layers.

Anchor	Level no	Load (kN)	Level (%)	Theoretical elongation (mm)	Measured elongation (mm)
Anchor 1, tension type	1	0	0	0.00	0.00
	2	100	11	9.91	4.96
	3	200	20	18.71	9.78
	4	300	30	27.97	15.01
	5	400	40	37.31	20.76
Anchor 2, compression type	1	0	0	0.00	0.00
	2	10	1	1.65	1.89
	3	30	2	3.30	4.56
	4	55	7	9.91	9.78
	5	100	14	19.82	14.28
	6	200	26	37.41	39.02
	7	300	39	55.94	69.83
	8	400	52	74.63	94.98
Anchor 3, compression type	1	0	0	0.00	0.00
	2	100	14	17.34	8.14
	3	200	26	32.72	13.19
	4	300	39	48.95	31.25
	5	400	52	65.30	51.71
Anchor 4, compression type	1	0	0	0.00	0.00
	2	10	1	1.44	1.89
	3	20	2	2.89	4.56
	4	55	7	8.67	9.78
	5	100	14	17.34	14.28
	6	200	26	32.74	16.65
	7	300	39	48.95	81.67
	8	400	52	65.30	115.54

Significant differences in elongation values were observed between tension and compression anchors. As shown in Table 8, the maximum elongation under a load of 400 kN was measured as 20.76 mm for tension type test anchor number 1, while for compression test anchor number 2, the value recorded under the same load was 94.98 mm. Similarly, compression anchors number 3 and 4 exhibited elongations of 51.71 mm and 115.64 mm, respectively, under a 400 kN load. The differences in elongation stem from variations in load transfer mechanisms. In tension-type anchors, the load concentrates in the bonded zone, potentially triggering crack formation, whereas in compression anchors, the load is transferred homogeneously through the structural element. These findings align with the results of the study conducted by Hsu and Chang^13^, where it was determined that the capacities of short tension-type anchors ranged between 143 and 231 kN/m, while short compression anchors had a capacity of approximately 200 kN/m. Furthermore, it was emphasized that as the length of tension-type anchors increases, their load-bearing capacities also increase, and that long compression anchors have a higher load-bearing capacity compared to tension type anchors.

**Fig. 16:**
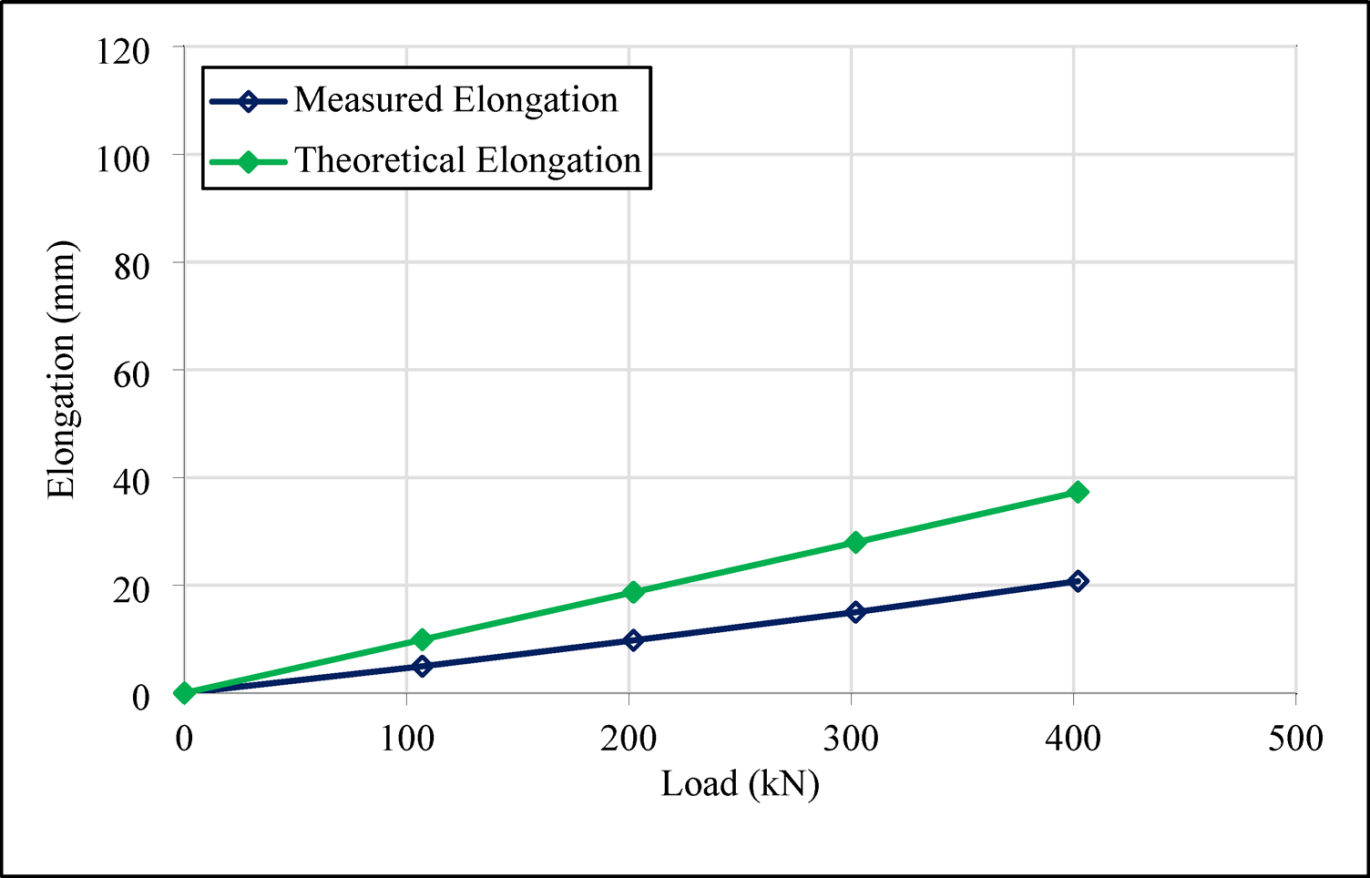
Load-elongation graph for number 1 tension type ground test anchor.

**Fig. 17:**
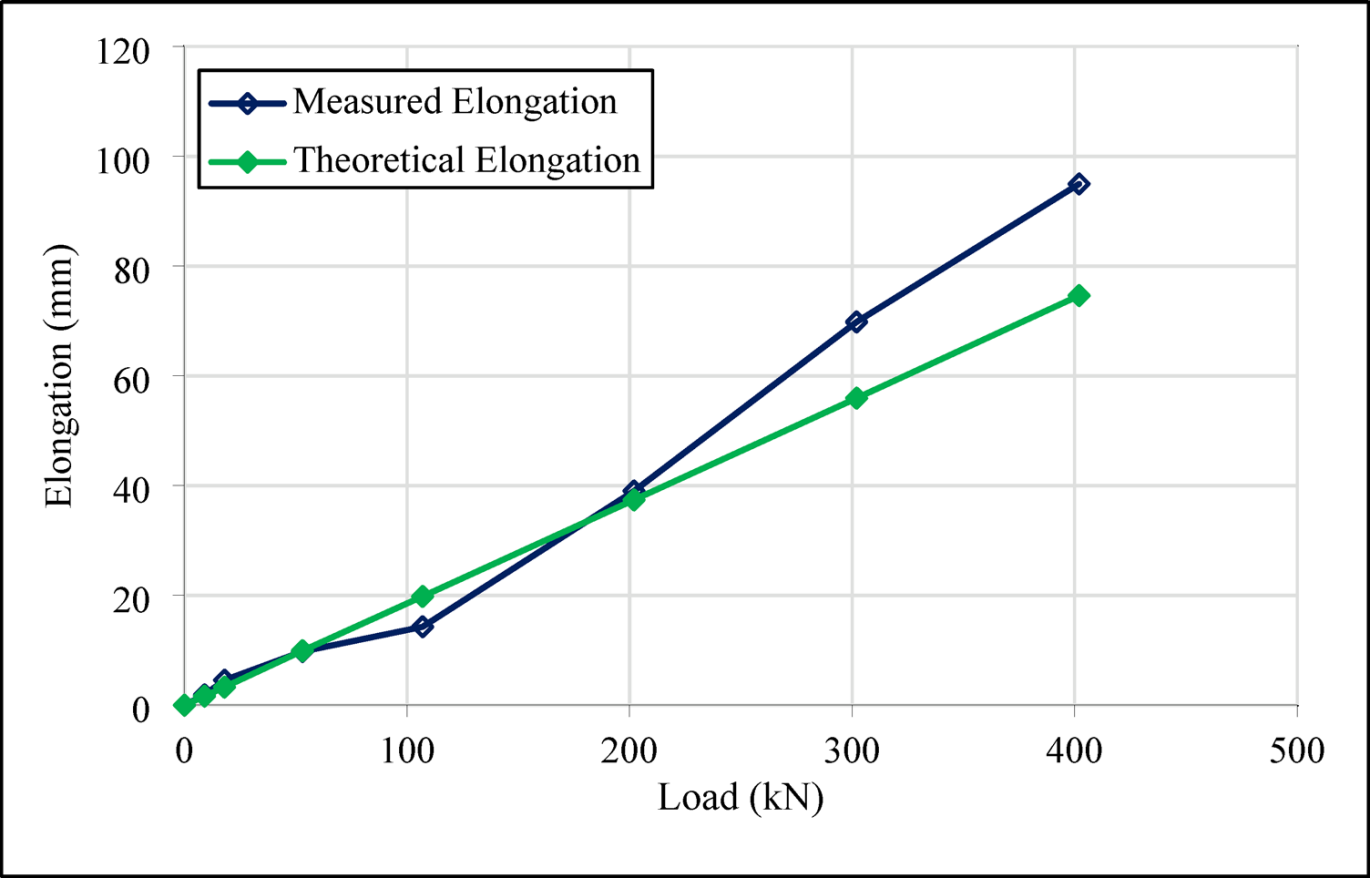
Load-elongation graph for number 2 compression type ground test anchor.

Figures 16 and 17 present the results of elongation values recorded at various load stages for the tension test anchor (Anchor 1) and the compression anchor (Anchor 2), respectively.

**Fig. 18:**
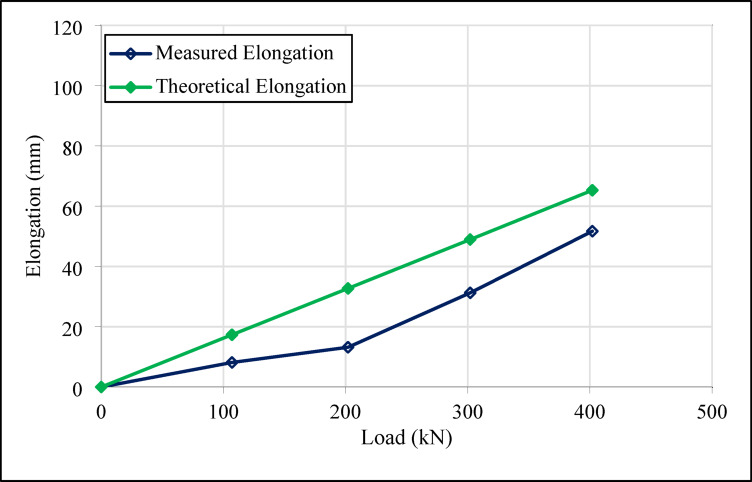
Load-elongation graph for number 3 compression type ground test anchor.

**Fig. 19:**
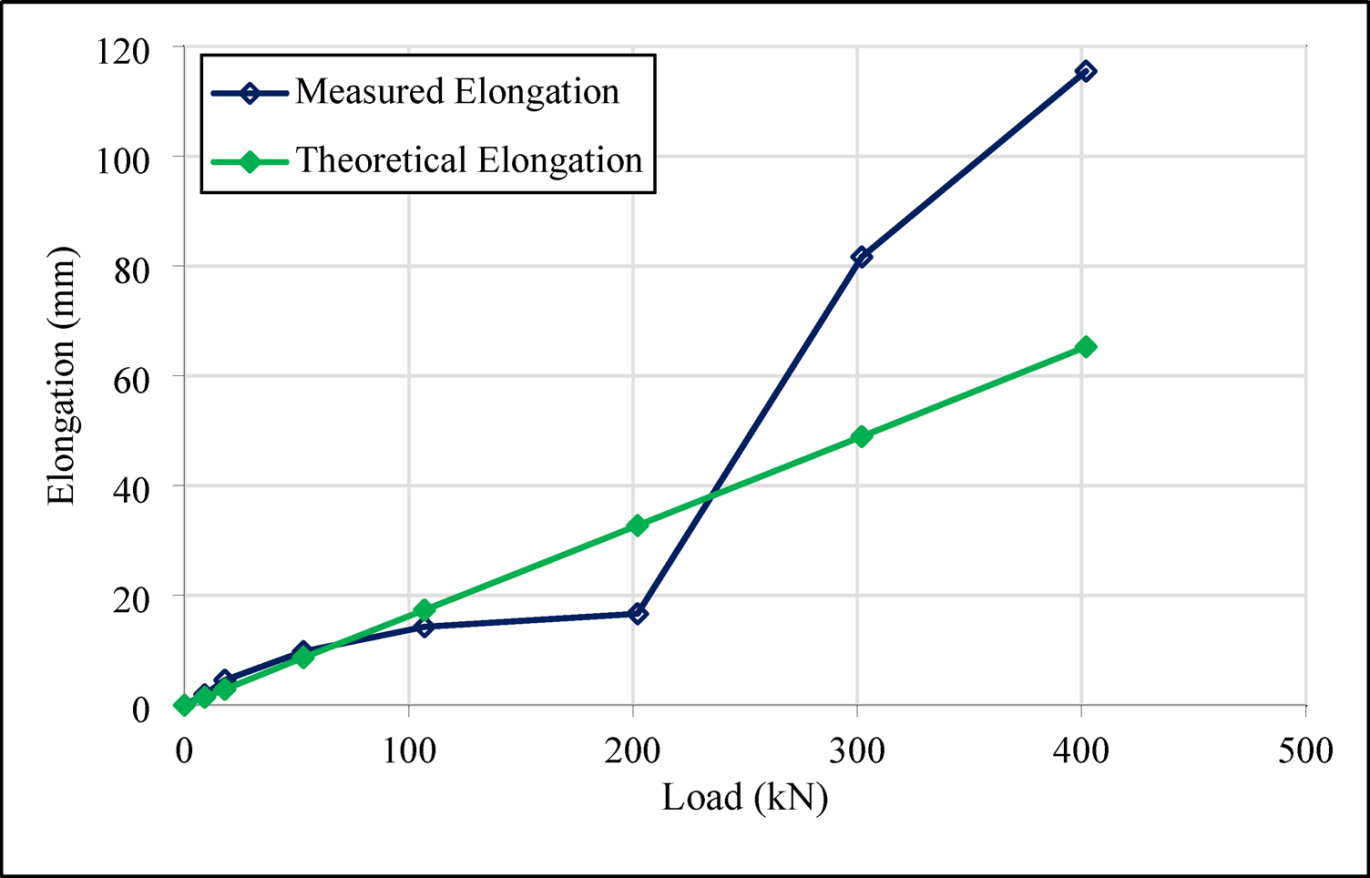
Load-elongation graph for number 4 compression type ground test anchor.

Figures 18 and 19 show the elongation results for the 14 m compression anchors (Anchors 3 and 4) at various load stages.

Figures 16, 17, 18 and 19 demonstrate that both tension and compression ground anchors exhibit reliable performance under high loading conditions in clay soils. The absence of failure in the steel tendons, grout-soil interface, or detachment in the bonded zone under the design load of 400 kN indicates that the system’s load-bearing capacity does not exceed the ultimate bearing capacity of the soil. These finding highlights that, even in relatively low-capacity soils such as clay, compression anchors can provide stability equivalent to tension anchors. Notably, the lack of detachment in the bonded zone for compression anchors 3 and 4, despite their shorter tendon lengths, reflects the superior performance of compression anchors in soil-anchor interaction and demonstrates that this performance is unaffected by variations in design dimensions.

The compression anchors showed relatively smaller elongations compared to tension anchors. Numerical stress distribution results further clarify this behavior: compression anchors mobilize shear resistance along a broader grout–soil interface, while a localized stress concentration develops at the tip, effectively engaging deeper soil layers. The strain profiles indicate that this combined side resistance and end-bearing mechanism reduces axial elongation compared to tension anchors, which rely mainly on bonded length adhesion. These mechanistic insights are consistent with the field test results, confirming that compression anchors provide a more efficient and stable load-transfer mechanism. In contrast, the lower elongation values in tension anchors indicate that the load concentration in the soil-anchor interaction is limited to a narrower region, which could potentially increase crack formation.

In conclusion, the field tests reveal that compression anchors effectively provide stability regardless of design dimensions and offer a broader load distribution compared to tension anchors. A study by Liu et al.^6^ proposed composite ground anchors by combining tension and compression anchors, and the performance of these systems was evaluated through field experiments. According to the field test results, the load-bearing capacity of a composite anchor was found to be 1.44 times greater than that of a tension anchor of the same length. These findings confirm that compression anchors provide higher load-bearing capacity and significant stability advantages compared to tension anchors.

## Conclusions

This study comprehensively examined the stability performance of compression and tension-type ground anchors used in deep excavations through both numerical analyses and field tests. The results demonstrate that compression anchors offer significant advantages over tension anchors, particularly in controlling deformations and ensuring stability under clay soil conditions, making them especially beneficial in densely built urban areas. Based on the findings from both field experiments and numerical analyses, the following conclusions can be drawn from the study:

- Numerical analyses showed that the average deformation in compression anchors was approximately 21% lower than that in tension anchors. At the final excavation level, the deformation in the grout body of compression anchors under ultimate loading was recorded as approximately 14% lower than in tension anchors. The higher deformation rates in tension anchors suggest that compression anchors are more effective in situations where deformation control is critical, especially in deep excavation projects.
- Compression anchors exhibit lower deformation rates even under high prestressing forces, offering a significant advantage in ensuring the safety of surrounding structures. Evaluations of safety factors further indicated that compression anchors are a more reliable choice compared to tension anchors.

Field tests revealed that, the maximum elongation measured in tension anchor 1 was 20.76 mm, while the corresponding value for compression anchor 2 reached 94.98 mm. Similarly, the elongation values for compression anchors 3 and 4 were recorded as 51.71 mm and 115.64 mm, respectively. The differences in elongation values were attributed to the homogeneity of the load transfer mechanism in compression anchors. In tension anchors, the load concentrated in the bonded zone, potentially causing cracks, whereas in compression anchors, the load was distributed over a broader area, maintaining stability.

In compression anchors, the load is directly transferred to the structural element at the end of the anchor hole through sheathed tendons, subjecting the grout to compression forces. This results in a more ductile behavior compared to their performance under tensile forces. Consequently, compression anchors, which transfer the load directly to the structural element without any failure in the grout, provide a more efficient soil-anchor interaction compared to tension anchors. This characteristic strongly supports the use of compression anchors, particularly in densely populated urban areas where controlling soil movements is critical.

This study provides valuable insights into the short-term stability performance of compression and tension anchors in deep excavation projects. It is recognized, however, that long-term factors such as sustained loading, creep, corrosion resistance, and durability play a decisive role in the service life of anchor systems. These aspects were beyond the scope of this work but represent important directions for future research to ensure comprehensive evaluation of anchor performance over their full lifecycle. Moreover, while this study focuses on pullout test results under clay soil conditions, it also highlights the need for additional field tests and evaluations in different soil types to better understand the behavior of compression anchors.

## Data Availability

The data used to support the findings of this study are available from the corresponding author upon reasonable request.
